# Characterization of T follicular helper cells in allogeneic normal pregnancy and PDL1 blockage-induced abortion

**DOI:** 10.1038/srep36560

**Published:** 2016-11-07

**Authors:** Weihong Zeng, Zhicui Liu, Siming Zhang, Jiabin Ren, Xiaoling Ma, Chuanmei Qin, Fuju Tian, Yan Zhang, Yi Lin

**Affiliations:** 1 Institute of Embryo-Fetal Original Adult Disease Affiliated to Shanghai Jiao Tong University School of Medicine, the International Peace Maternity & Child Health Hospital, Shanghai Jiao Tong University School of Medicine, Shanghai 200030, P. R. China; 2 Department of Dermatology, Ruijin Hospital, Shanghai Jiaotong University School of Medicine, Shanghai 200025, P. R. China; 3 Department of Obstetrics and Gynecology, Renji Hospital, Shanghai Jiaotong University School of Medicine, Shanghai 200127, P. R. China; 4 Department of Obstetrics and Gynecology, Renmin Hospital of Wuhan University, Wuhan 430060, P. R. China

## Abstract

A deeper understanding of the immunological events during pregnancy will provide novel insights into the pathogenesis of pregnancy complications. The fundamental function of T follicular helper (Tfh) cells is to provide cognate help to B cells. Dysregulations of Tfh-cell function and/or development can result in various immunological diseases. However, the role and characteristics of Tfh cells during pregnancy remain unknown. Herein, an allogeneic-normal-pregnant mouse model was used, and we found that the CD4^+^ T cells residing at the uterus and placenta (UP) displayed a Tfh-like phenotype; and the UP-derived CD4^+^CXCR5^hi^PD-1^hi^ and CD4^+^CXCR5^hi^ICOS^hi^ Tfh cells, which showed a memory/activation phenotype, reached their peak at mid-pregnancy. These Tfh cells were located abundantly in the uterus at mid-pregnancy, but greatly increased in the placenta at late-pregnancy. Furthermore, increased foetal resorption by PDL1 blockade correlated with enhanced accumulation of Tfh cells and upregulated expressions of ICOS and PD-1 on these cells. Collectively, our findings are the first to indicate that an adequate and balanced accumulation of Tfh cells during gestation is likely to help maintaining a successful pregnancy, whereas an excessively high level of these cells could lead to abortion.

As a vital factor for species maintenance, reproduction is an extremely complicated biological process^1^. The question of how placental mammals with normal immune function can successfully carry the semi-allogeneic foetus and placenta to full term without immune rejection has intrigued reproductive biologists and immunologists for more than 60 years^1^,^2^. The immune cells residing at the maternal-foetal interface are thought to have many important roles in the maintenance of a normal pregnancy, and a deeper understanding of the immunological events during pregnancy will provide insights into the pathogenesis of many pregnancy complications, such as spontaneous abortion, intrauterine growth restriction, preterm birth and preeclampsia^3^.

Upon encountering antigens presented by antigen-presenting cells (APCs) and driven by lineage-specific transcription factors and cytokines, naive CD4^+^ T helper (Th) cells can differentiate into different effector subsets, such as Th1, Th2, Th17, T regulatory (Treg) and T follicular helper (Tfh) cells^4^. Th1 cells are characterized by their production of IFN-γ and play an important role in cellular immunity against intracellular microorganisms, and Th17 cells are characterized by producing IL-17 and have important functions in clearance of extracellular bacteria and fungi; both of these cells are responsible for acute allograft rejection and are involved in the pathogenesis of pregnancy complications such as spontaneous abortion and preeclampsia^5^,^6^,^7^,^8^. However, Th2 cells which are characterized by producing Th2 cytokines (IL-4, IL-5, IL-13) and are required for humoral immunity to control extracellular pathogens, together with Treg cells that are characterized by expressing the forkhead transcription factor Foxp3 and having an essential role in maintenance of immune homeostasis, are thought to be responsible for allograft tolerance and be involved in inducing maternal-foetal immune tolerance and maintaining a successful pregnancy^5^,^6^,^7^,^8^.

As a novel and distinct lineage of CD4^+^ T cells, Tfh cells were first identified in human lymphoid tissues (tonsils) that express CXC chemokine receptor 5 (CXCR5) and have B cell helper function in 2000^9^,^10^. In contrast to other CD4^+^ T cell lineages, Tfh cells express low levels of cytokines (IFN-γ, IL-4, IL-17 and TGF-β) and transcription factors (T-bet, GATA3, RORγt and Foxp3), which are characteristics of Th1, Th2, Th17 and CD4^+^ Treg cells, respectively^11^. Furthermore, Tfh cells display a unique expression profile of effector molecules, including high levels of surface receptors such as inducible costimulatory molecule (ICOS), programmed death-1 (PD-1), CD40 ligand (CD40L), OX40, IL-21R, B- and T-lymphocyte attenuator (BTLA) and CD84, the cytokine IL-21, and the master transcription factor B-cell lymphoma 6 (BCL-6), which are critical for the development and functionality of Tfh cells^4^,^11^,^12^. The fundamental function of Tfh cells is to provide cognate help to B cells, a pivotal event for the generation of a T cell-dependent B cell response^11^. Growing evidence suggests that dysregulations of Tfh-cell function and/or development can result in various immune diseases, such as autoimmunity, immunodeficiency, malignancy and allograft rejection^11^,^13^,^14^.

However, the role and characteristics of Tfh cells during pregnancy have never been reported. Here, an allogeneic-normal-pregnant mouse model was used to show that the CD4^+^ T cells residing at the uterus and placenta (UP) displayed a Tfh-like phenotype, and the UP-derived Tfh cells displaying a memory/activation phenotype were preferentially enriched at mid-pregnancy. In addition, these Tfh cells were located abundantly in the uterus at mid-pregnancy, but greatly increased in the placenta at late-pregnancy. Moreover, increased foetal resorption by PDL1 blockade did not correlate with the cellularity of maternal total CD4^+^ T cells, but correlated with enhanced Tfh-cell accumulation and upregulated ICOS and PD-1 expression on Tfh cells. Taken together, our findings indicated that Tfh cells might be involved in the maintenance of a successful allogeneic pregnancy.

## Results

### UP-derived CD4^+^ T cells display Tfh-like and memory/activation phenotypes

**Figure 1:**
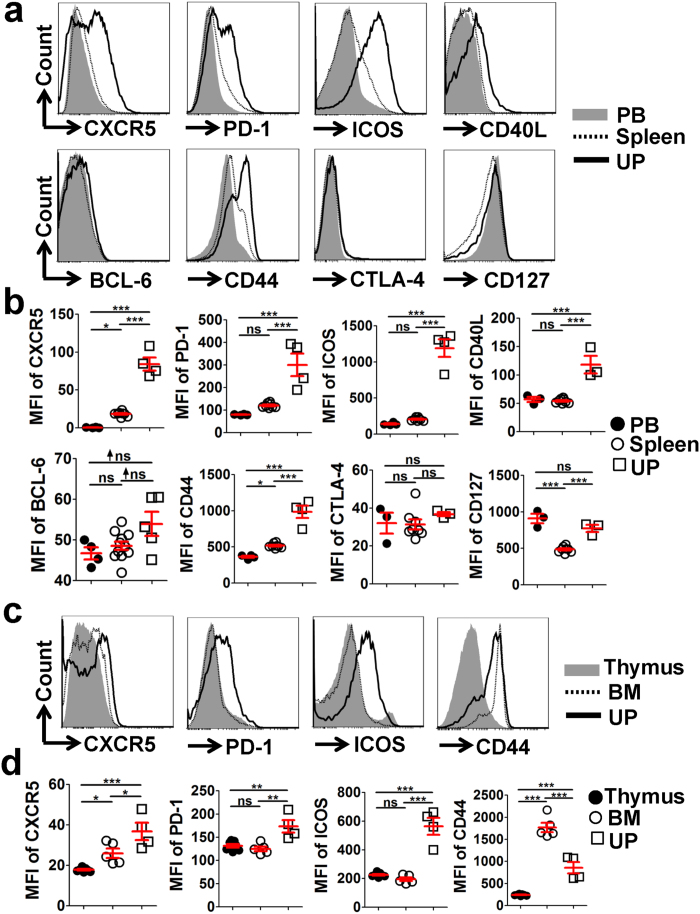
UP-derived CD4^+^ T cells display Tfh-like and memory/activation phenotypes at mid-pregnancy. Adult female BALB/c mice were mated with male C57BL/6 mice and embryonic day 0.5 (E0.5) was defined as the day of vaginal-plug discovery. **(a–d)** Representative flow cytometric histograms (**a**,**c**) and cumulative data (**b**,**d**) illustrating the molecular expression of the indicated proteins on/in CD4^+^ T cells derived from the PB, spleen and UP (**a**,**b**), as well as from the thymus, BM and UP (**c**,**d**), of maternal mice on E11.5. Each symbol reflects the data from a single mouse (n ≥ 3 mice per group) and the data are representative of at least three independent experiments. The cells are gated in CD4^+^ T cells. Geometric MFI values were calculated using FlowJo 7.6.1 software and the data were assessed statistically using one-way ANOVA followed by Tukey’s post-tests. PB: peripheral blood; UP: uterus and placenta; BM: bone marrow; MFI: mean fluorescent intensity; ns: not significant; *p < 0.05; **p < 0.01; ***p < 0.001.

To investigate whether the CD4^+^ T cells residing at the UP are phenotypically similar to those derived from other lymphoid tissues during allogeneic-normal-pregnancy, adult female BALB/c mice were mated with male C57BL/6 mice and embryonic day 0.5 (E0.5) was defined as the day of vaginal-plug discovery. The expression level of characteristic proteins on/in CD4^+^ T cells was determined using multi-color flow cytometry at mid-pregnancy (on E11.5). Interestingly, we found that the levels of Tfh-associated surface molecules including CXCR5, PD-1, ICOS and CD40L, and the memory/activation marker CD44, were remarkably higher on the CD4^+^ T cells derived from the UP than those from the peripheral blood (PB) and spleen, whereas no significant differences were observed in the expressions of CTLA-4 and CD127 on CD4^+^ T cells between the PB and UP (Fig. 1a,b and Supplementary Fig. 1a,b). In addition, the level of BCL-6, a key transcription factor of Tfh cells, was also slightly (non-significantly) higher in CD4^+^ T cells derived from the UP than those from the PB and spleen (Fig. 1a,b). Furthermore, significantly higher levels of Tfh-associated molecules CXCR5, PD-1 and ICOS were observed on the CD4^+^ T cells derived from the UP compared with those from the bone marrow (BM) and thymus (Fig. 1c,d). CD4^+^ T cells derived from the UP showed a dramatically higher level of the memory/activation marker CD44 than those derived from the thymus (Fig. 1c,d and Supplementary Fig. 1c,d) as well. These data demonstrated that UP-derived CD4^+^ T cells display Tfh-like and memory/activation phenotypes in the normal allogeneic pregnant mice at mid-pregnancy.

### CD4^+^CXCR5^hi^PD-1^hi^ and CD4^+^CXCR5^hi^ICOS^hi^ Tfh cells are preferentially accumulated in the UP

**Figure 2:**
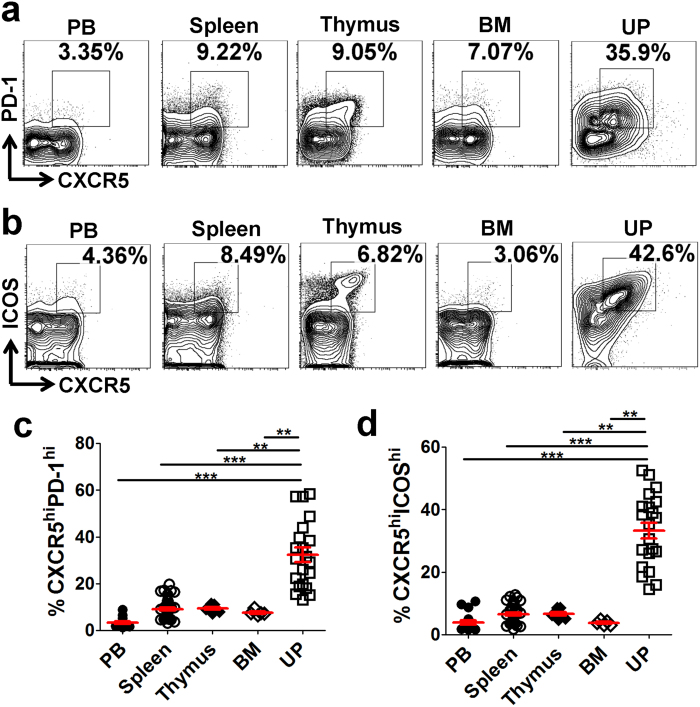
CD4^+^CXCR5^hi^PD-1^hi^ and CD4^+^CXCR5^hi^ICOS^hi^ Tfh cells are preferentially accumulated in the UP. **(a**–**d)** Representative flow cytometric plots and cumulative data illustrating the percentages of CXCR5^hi^PD-1^hi^ (**a**,**c**) and CXCR5^hi^ICOS^hi^ (**b**,**d**) populations among CD4^+^ T cells in the PB, spleen, thymus, BM and UP of maternal mice on E11.5. Each symbol reflects the data from a single mouse (n ≥ 6 mice per group) and the data are representative of two independent experiments. The cells are gated in CD4^+^ T cells. Data were assessed statistically using the Kruskal-Wallis test followed by Dunns multiple-comparison test. PB: peripheral blood; BM: bone marrow; UP: uterus and placenta; hi: high; **p < 0.01; ***p < 0.001.

Tfh cells have been defined as CXCR5^hi^PD-1^hi^ or CXCR5^hi^ICOS^hi^ populations gated in CD4^+^ T cells^15^,^16^,^17^. The above data showed that UP-derived CD4^+^ T cells had the highest expression level of Tfh-associated molecules (Fig. 1 and Supplementary Fig. 1). Therefore, the proportion of Tfh cells was further compared among different lymphoid tissues on E11.5. We observed that the proportions of CD4^+^CXCR5^hi^PD-1^hi^ and CD4^+^CXCR5^hi^ICOS^hi^ Tfh cells derived from the UP were strikingly higher than those from the PB, spleen, thymus and BM (Fig. 2a–d), indicating that these Tfh cells are dominantly enriched in the UP.

### UP-derived Tfh cells show high levels of Tfh-associated molecules and display a memory/activation phenotype

**Figure 3:**
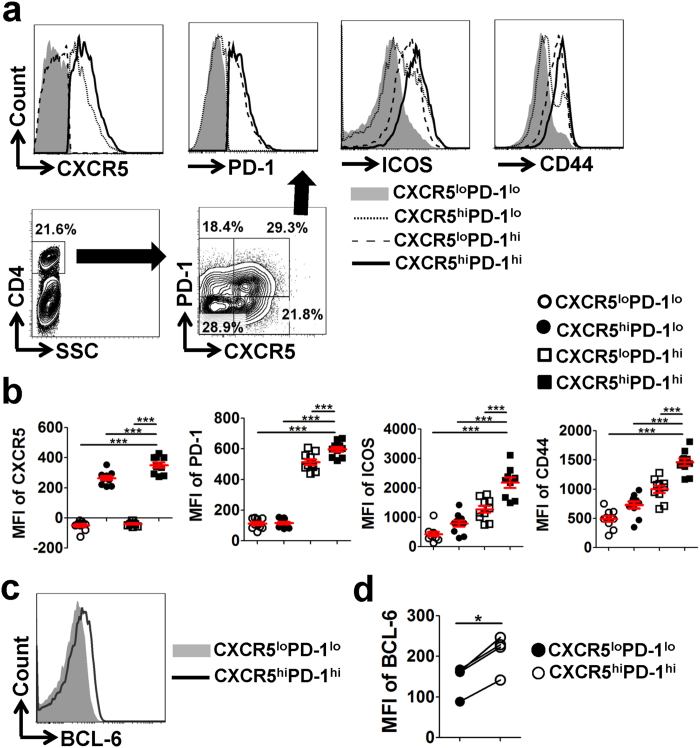
UP-derived CD4^+^CXCR5^hi^PD-1^hi^ Tfh cells show high levels of Tfh-associated molecules and display a memory/activation phenotype. (**a**) CD4^+^ T cells were gated from the lymphocytes in the UP of pregnant mice on E11.5, and a schematic diagram illustrates the strategy used to analyse the molecular expressions of the indicated proteins among CD4^+^CXCR5^lo^PD-1^lo^, CD4^+^CXCR5^hi^PD-1^lo^, CD4^+^CXCR5^lo^PD-1^hi^ and CD4^+^CXCR5^hi^PD-1^hi^ cells. (**b**) Comparisons of CXCR5, PD-1, ICOS and CD44 expression among the indicated CD4^+^ T-cell subsets derived from the UP on E11.5. (**c**,**d**) Intra-nuclear expression of BCL-6 was detected according to the Foxp3 staining protocol, and representative flow histograms and cumulative data illustrate BCL-6 level within CD4^+^CXCR5^lo^PD-1^lo^ and CD4^+^CXCR5^hi^PD-1^hi^ cells derived from the UP of pregnant mice on E11.5. Each symbol reflects the data from a single mouse (n ≥ 4 mice per group) and the data are representative of at least three independent experiments. Geometric MFI values were calculated using FlowJo 7.6.1 software and the data were assessed statistically using one-way ANOVA followed by Tukey’s post-tests (**b**) or Wilcoxon matched pairs test (**d**). UP: uterus and placenta; MFI: mean fluorescent intensity; hi: high; lo, low; **p < 0.05; ***p < 0.001.

To characterize the Tfh cells residing at the UP, CD4^+^ T cells were gated from the leukocytes in the UP at mid-pregnancy, and those cells were subsequently divided into four sub-populations based on their expressions of CXCR5 and PD-1 (or ICOS), and the abundance of characteristic proteins was further investigated among these sub-populations (Fig. 3a and Supplementary Fig. 2a), as previous studies performed^18^,^19^,^20^. Both CD4^+^CXCR5^hi^PD-1^hi^ and CD4^+^CXCR5^hi^ICOS^hi^ Tfh cells expressed the highest levels of Tfh-associated molecules CXCR5, PD-1 and ICOS, as well as the memory/activation marker CD44, compared with the other three subsets (Fig. 3a,b and Supplementary Fig. 2). In addition, CD4^+^CXCR5^hi^PD-1^hi^ Tfh cells showed a significantly higher level of Tfh-cell key transcriptional factor BCL-6 than CD4^+^CXCR5^lo^PD-1^lo^ cells (Fig. 3c,d). Taken together, these data demonstrated that UP-derived CD4^+^CXCR5^hi^PD-1^hi^ and CD4^+^CXCR5^hi^ICOS^hi^ Tfh cells display high levels of Tfh-associated molecules and a memory/activation phenotype.

### UP-derived Tfh cells are dominantly enriched at mid-gestation and display a distinct phenotype during pregnancy

**Figure 4:**
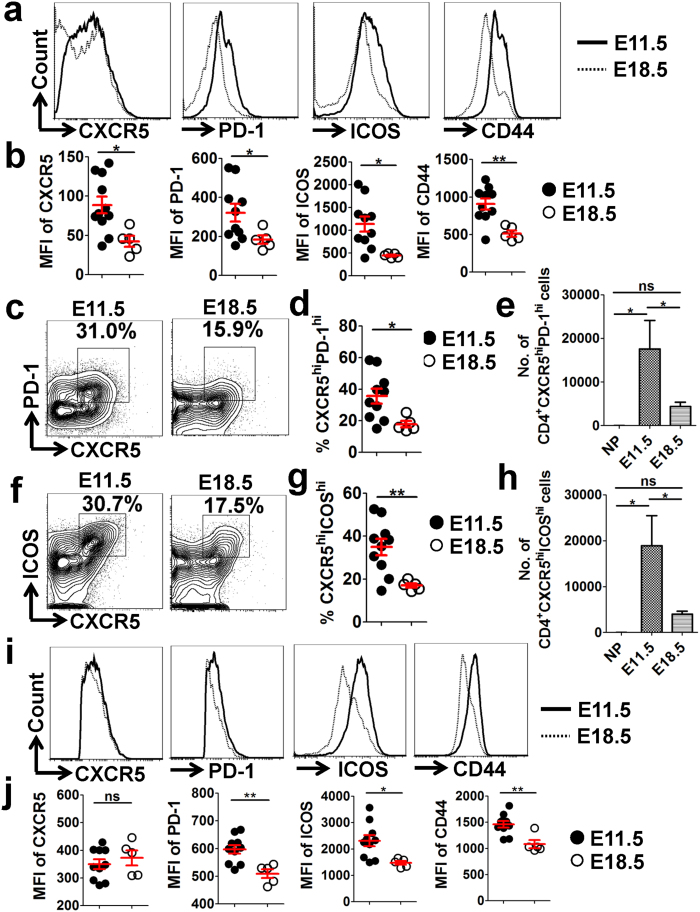
UP-derived Tfh cells are dominantly enriched at mid-gestation and display a distinct phenotype during pregnancy. (**a**–**h**) Representative flow cytometric histograms (or plots) and cumulative data illustrating the molecular expression of CXCR5, PD-1, ICOS and CD44 on CD4^+^ T cells (**a**,**b**), as well as the percentage (**c,d,f,g**) and absolute number (**e**,**h**) of CD4^+^CXCR5^hi^PD-1^hi^ and CD4^+^CXCR5^hi^ICOS^hi^ Tfh cells, derived from the UP of pregnant mice at the indicated time or from the uterus of NP mice. (**i**,**j)** Representative flow cytometric histograms (**i**) and cumulative data (**j**) illustrating the expression level of CXCR5, PD-1, ICOS and CD44 on CD4^+^CXCR5^hi^PD-1^hi^ Tfh cells derived from the UP of pregnant mice on E11.5 and E18.5, respectively. Each symbol reflects the data from a single mouse (n ≥ 5 mice per group) and the data are representative of at least two independent experiments. The cells in Fig. a, c and f are gated in CD4^+^ T cells, and in Fig. i are gated in CD4^+^CXCR5^hi^PD-1^hi^ cells. Geometric MFI values were calculated with FlowJo 7.6.1 software and the statistical analysis was performed using an unpaired Student’s *t*-test (**b**,**d**,**g**,**j**) or the Kruskal-Wallis test followed by Dunns multiple-comparison test (**e**,**h**). UP: uterus and placenta; MFI: mean fluorescent intensity; No.: number; NP: non-pregnant; hi: high; ns: not significant; *p < 0.05; **p < 0.01.

The dynamic change of Tfh cells was then further monitored during pregnancy. Our analysis revealed that the UP-derived CD4^+^ T cells expressed significantly higher levels of Tfh-associated molecules CXCR5, PD-1 and ICOS, as well as the memory/activation marker CD44, on E11.5 compared with E18.5 (Fig. 4a,b and Supplementary Fig. 3). Moreover, higher proportions and absolute numbers of CD4^+^CXCR5^hi^PD-1^hi^ and CD4^+^CXCR5^hi^ICOS^hi^ Tfh cells were observed in the UP on E11.5 than on E18.5 (Fig. 4c–h), whereas very few Tfh cells were detected in the uterus of non-pregnant (NP) mice (Fig. 4e,h). In addition, UP-derived CD4^+^CXCR5^hi^PD-1^hi^ Tfh cells had strikingly higher levels of PD-1, ICOS and CD44 on E11.5 compared with E18.5 (Fig. 4i,j). However, the expression of Tfh-associated molecules CXCR5, PD-1 and ICOS on CD4^+^ T cells, together with the proportion and concentration (absolute number per gram) of CD4^+^CXCR5^hi^PD-1^hi^ and CD4^+^CXCR5^hi^ICOS^hi^ Tfh cells, were not significantly changed in the maternal spleen (Supplementary Fig. 4) and thymus (data not shown) during pregnancy. Taken together, these data suggested that Tfh cells residing at the UP accumulate preferentially at the mid-stage of gestation and display a distinct phenotype during pregnancy.

### Distribution of Tfh cells at the uterus and placenta

**Figure 5:**
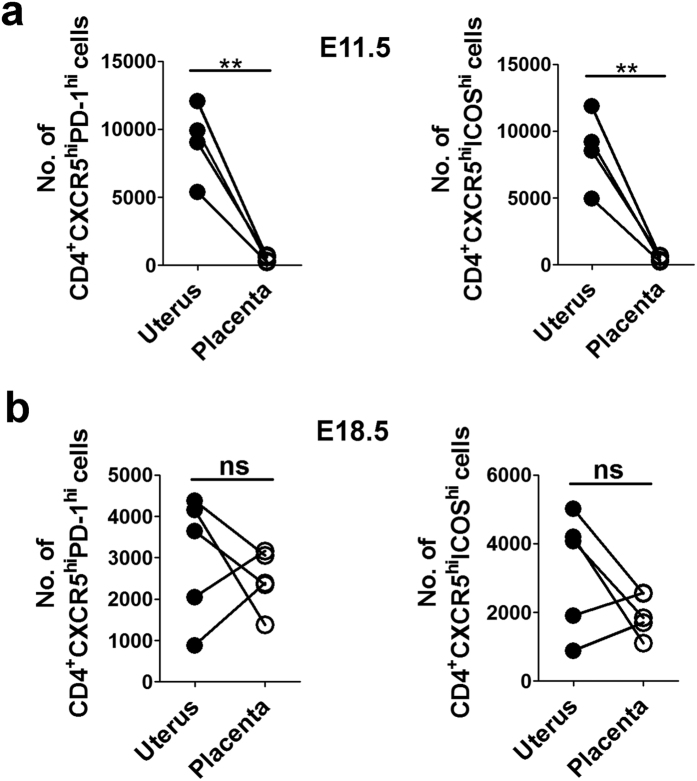
Distribution of Tfh cells at the uterus and placenta. The uterus and placenta were isolated separately from C57BL/6-mated BALB/c females, and the absolute numbers of CD4^+^CXCR5^hi^PD-1^hi^ and CD4^+^CXCR5^hi^ICOS^hi^ Tfh cells were determined by flow cytometry on E11.5 (**a**) and E18.5 (**b**). Each symbol reflects the data from a single mouse (n ≥ 4 mice per group) and the data are representative of at least two independent experiments. Statistical analysis was performed using paired Student’s *t*-test. No.: number; hi: high; ns: not significant; **p < 0.01.

To further investigate the distribution pattern of Tfh cells at the UP during allogeneic pregnancy, the uterus and placenta were isolated separately from C57BL/6-mated BALB/c females, and the cellularity of Tfh cells was determined by flow cytometry on E11.5 and E18.5, respectively. At mid-pregnancy (E11.5), both CD4^+^CXCR5^hi^PD-1^hi^ and CD4^+^CXCR5^hi^ICOS^hi^ Tfh cells were abundantly located in the uterus, but were almost absent in the placenta (Fig. 5a). However, at late-pregnancy (E18.5), the absolute number of Tfh cells greatly increased in the placenta, reaching a comparable level to that found in the uterus (Fig. 5b).

### Increased foetal resorption by PDL1 blockade does not correlate with the cellularity of total CD4^+^ T cells

**Figure 6:**
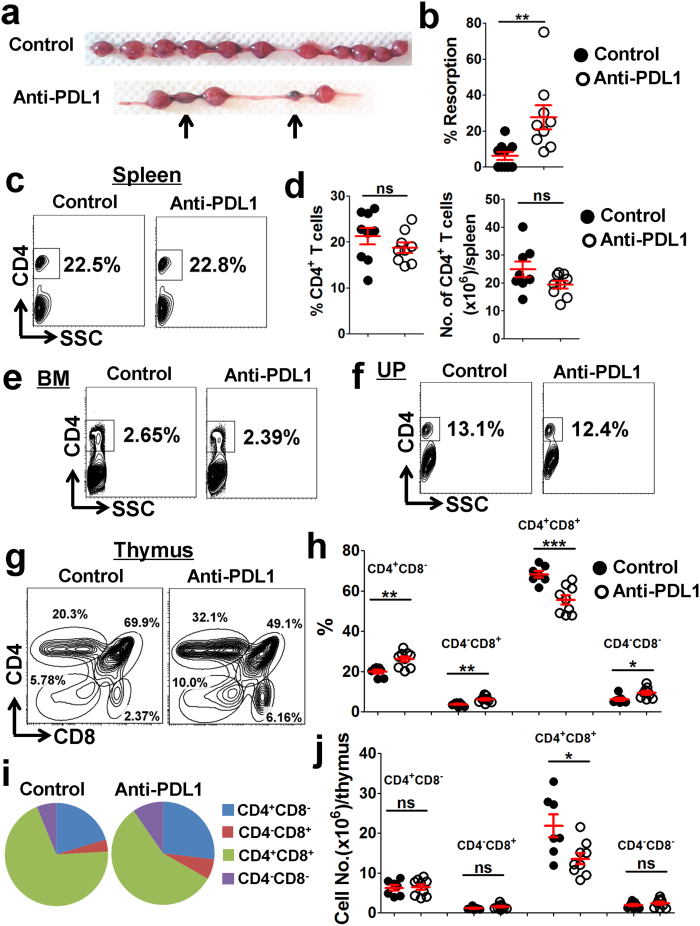
Effects of PDL1 blockade on foetal resorption, and on the proportion, cellularity and maturation of maternal CD4^+^ T cells. Adult BALB/c mice were mated with C57BL/6 mice, and the pregnant females were then injected intraperitoneally with anti-mouse PDL1 blocking mAb at a dose of 250 μg on E5.5 and E8.5, respectively. Both the control and PDL1-blocked mice were sacrificed on E11.5. **(a)** Representative diagrams illustrate the whole uterus (containing embryos and placentas) of the control and PDL1-blocked mice, and resorbed embryos are indicated by arrows. **(b)** Resorption rate. **(c**,**d)** Representative flow cytometric plots (**c**) and cumulative data (**d**) illustrating the percentage and absolute number of CD4^+^ T cells in the maternal spleen. **(e**,**f)** Representative flow cytometric plots illustrating the percentage of CD4^+^ T cells in the maternal BM (**e**) and UP (**f**). **(g**–**i)** Representative flow cytometric plots (**g**), bar graphs (**h**) and pie charts (**i**) displaying the percentages of indicated cells in the maternal thymus from the control and PDL1-blocked mice. **(j)** The absolute number of indicated cells in the maternal thymus. Each symbol reflects the data from a single mouse (n ≥ 8 mice per group) and the data are combined from four independent experiments. The cells are gated from lymphocytes. The data were assessed statistically using the Mann-Whitney U test (**a**) or unpaired Student’s *t*-test (**d**,**h**,**j**). BM: bone marrow; UP: uterus and placenta; No.: number; ns: not significant; *p < 0.05; **p < 0.01; ***p < 0.001.

To establish an induced-abortion murine model, adult BALB/c mice were mated with C57BL/6, and pregnant females were injected intraperitoneally with anti-mouse PDL1 blocking monoclonal antibody (mAb) on E5.5 and E8.5, respectively. Both the IgG-treated controls and PDL1-blocked pregnant mice were sacrificed on E11.5. In line with previous reports^21^,^22^,^23^, we observed that blockage of PDL1 led to increased foetal resorption (Fig. 6a,b). However, the body-, spleen- and thymus-weight; the proportion of CD4^+^ T cells in the spleen, BM and UP; as well as the absolute numbers of the splenocytes, thymocytes and splenic CD4^+^ T cells; were not significantly different between the control and PDL1-blocked maternal mice (Fig. 6c–f and Supplementary Fig. S5). Although PDL1 blockage increased the proportions of thymic CD4^+^CD8^−^, CD4^−^CD8^+^ and CD4^−^CD8^−^ cells, the absolute numbers of these cells were not significantly different from the controls (Fig. 6g–j). These data indicated that increased foetal resorption by PDL1 blockade does not correlate with the cellularity of total CD4^+^ T cells.

### Increased foetal resorption by PDL1 blockade correlates with enhanced accumulation of Tfh cells

**Figure 7:**
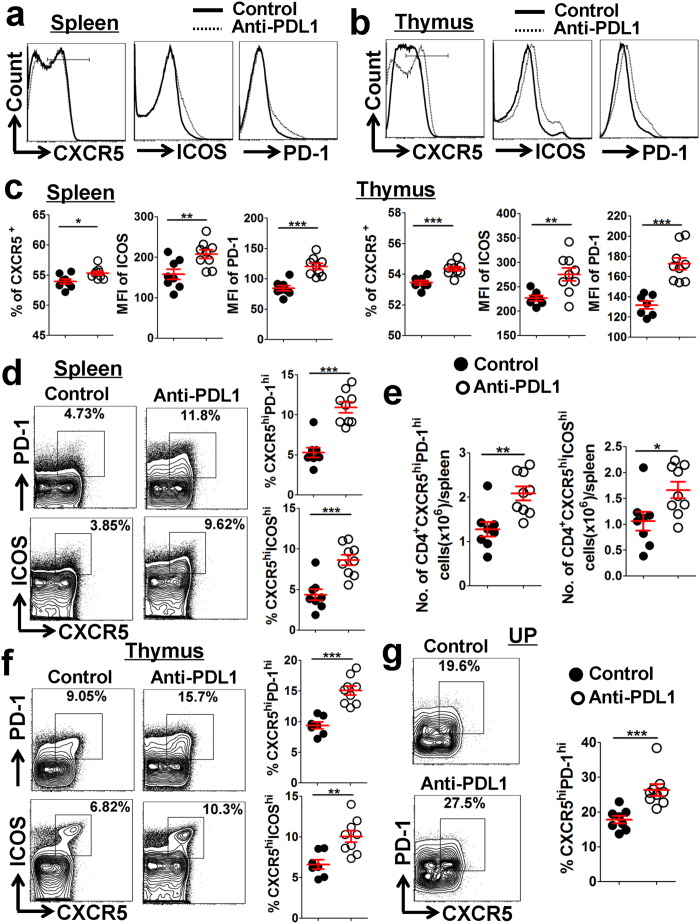
Increased foetal resorption by PDL1 blockade correlates with an increase in Tfh cells. **(a**–**c)** Representative flow cytometric histograms (**a**,**b**) and cumulative data (**c**) illustrating the molecular expression of CXCR5, ICOS and PD-1 on CD4^+^ T cells in the maternal spleen and thymus from the control and PDL1 blockade mice. **(d**–**f)** Representative flow cytometric plots and cumulative data illustrating the percentages of CXCR5^hi^PD-1^hi^ and CXCR5^hi^ICOS^hi^ populations among CD4^+^ T cells in maternal spleen (**d**) and thymus (**f**), as well as the absolute numbers of CD4^+^CXCR5^hi^PD-1^hi^ and CD4^+^CXCR5^hi^ICOS^hi^ Tfh cells in each spleen (**e**), of the control and PDL1-blocked mice. **(g)** Comparison of the percentage of CXCR5^hi^PD-1^hi^ population among UP-derived CD4^+^ T cells between the control and PDL1 blockade mice. Each symbol reflects the data from a single mouse (n ≥ 8 mice per group) and the data are combined from four independent experiments. The cells are gated in CD4^+^ T cells. Geometric MFI values were calculated using FlowJo 7.6.1 software and the data were assessed statistically using an unpaired Student’s *t*-test. MFI: mean fluorescent intensity; UP: uterus and placenta; hi: high; No.: number; *p < 0.05; **p < 0.01; ***p < 0.001.

Interestingly, our data showed that both splenic and thymic CD4^+^ T cells expressed strikingly higher levels of Tfh-associated molecules CXCR5, ICOS and PD-1 in PDL1-blocked maternal mice than the controls (Fig. 7a–c and Supplementary Fig. S6a,b). Moreover, the proportions of CD4^+^CXCR5^hi^PD-1^hi^ and CD4^+^CXCR5^hi^ICOS^hi^ Tfh cells in the spleen, thymus, BM and UP, as well as the absolute numbers of these Tfh cells in the spleen and thymus, were remarkably higher in the PDL1-blocked mice (Fig. 7d–g and Supplementary Fig. S6c,d). These data suggested that increased foetal resorption by PDL1 blockade correlates with enhanced accumulation of Tfh cells.

### PDL1 blockade upregulates ICOS and PD-1 expression on Tfh cells

**Figure 8:**
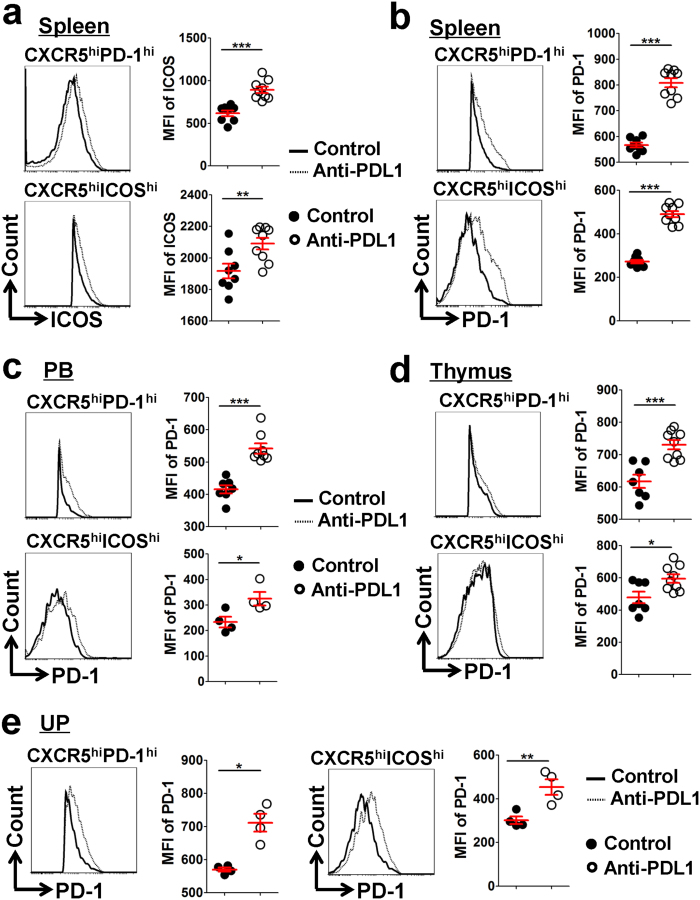
PDL1 blockade upregulates ICOS and PD-1 expression on Tfh cells. **(a**,**b)** Representative flow cytometric histograms and cumulative data illustrating ICOS (**a**) and PD-1 (**b**) expression on CD4^+^CXCR5^hi^PD-1^hi^ and CD4^+^CXCR5^hi^ICOS^hi^ Tfh cells in the maternal spleens of the control and PDL1-blocked mice. **(c**–**e)** Representative flow cytometric histograms and cumulative data illustrating PD-1 expression on CD4^+^CXCR5^hi^PD-1^hi^ and CD4^+^CXCR5^hi^ICOS^hi^ Tfh cells in the maternal PB (**c**), thymus (**d**) and UP (**e**) of the control and PDL1-blocked mice. Geometric MFI values were calculated with FlowJo 7.6.1 software. Each symbol reflects the data from a single mouse (n ≥ 4 mice per group) and the data were assessed statistically using an unpaired Student’s *t*-test (**a**–**d**) or the Mann-Whitney U test (**e**). The cells are gated in CD4^+^CXCR5^hi^PD-1^hi^ or CD4^+^CXCR5^hi^ICOS^hi^ cells as indicated. MFI: mean fluorescent intensity; PB: peripheral blood; UP: uterus and placenta; hi: high; *p < 0.05; **p < 0.01; ***p < 0.001.

Finally, the effect of PDL1 blockade on the molecular expression of characteristic proteins on Tfh cells was further investigated. Our analysis revealed that the CD4^+^CXCR5^hi^PD-1^hi^ and/or CD4^+^CXCR5^hi^ICOS^hi^ Tfh cells expressed strikingly higher levels of ICOS and PD-1 in the maternal spleen and BM from the PDL1-blocked mice compared with the controls (Fig. 8a,b and Supplementary Fig. S7). Additionally, the PB, thymus and UP from PDL1-blocked mice showed a significantly higher level of PD-1 on CD4^+^CXCR5^hi^PD-1^hi^ and CD4^+^CXCR5^hi^ICOS^hi^ Tfh cells than the controls (Fig. 8c–e). These data indicated that increased foetal resorption by PDL1 blockade correlates with upregulated ICOS and PD-1 expressions on Tfh cells.

## Discussion

As a potent chemokine, CXCL13 [chemokine (C-X-C motif) ligand 13] is mainly produced by monocytes, macrophages, dendritic cells and lymphocytes, and has been detected in serum, normal lymphoid or inflammatory tissues^24^,^25^. CXCR5, the only known receptor for CXCL13, is expressed by mature B cells, Tfh cells and immature dendritic cells^26^,^27^. Recently, a high level of CXCL13 was detected in the maternal serum, umbilical cord blood and amniotic fluid of pregnant women^28^, and another group showed that the human decidual stromal cells (DSCs) from the elective termination of pregnancy secreted this chemokine, suggesting that DSCs are involved in CXCL13 secretion during human pregnancy^29^. Our data also showed that the CXCL13 level in murine serum was greatly elevated during allogeneic normal pregnancy (Supplementary Fig. 8). In addition, CD4^+^ T cells residing at the UP displayed a high level of the CXCL13 receptor CXCR5 (Fig. 1 and Supplementary Fig. S1), and both UP-derived CD4^+^CXCR5^hi^PD-1^hi^ and CD4^+^CXCR5^hi^ICOS^hi^ Tfh cells were preferentially enriched at mid-pregnancy (Figs 2 and 4). Purified CD4^+^ T cells were also sorted from the deciduas (dCD4^+^ T) and peripheral blood (pCD4^+^ T) of healthy pregnant women at the first trimester, and we demonstrated that dCD4^+^ T cells up-regulated the mRNA levels of Tfh-associated molecules *CXCR5*, *PD-1*, *ICOS*, *CD40L*, *OX40*, etc., and down-regulated *CCR7* and *c-Maf*, compared with the pCD4^+^ T cells (unpublished data).

Differentiation of CD4^+^ T-cell effector subsets is driven by lineage-specific transcription factors, and Tfh-cell differentiation is regulated by the transcription factor BCL-6^30^. Enhanced expression of BCL-6 promotes Tfh-cell differentiation through potential mechanisms by suppressing the lineage-specific transcriptional factors T-bet, GATA3, RORγt and Foxp3, which are characteristics of Th1, Th2, Th17 and CD4^+^ Treg cells, respectively^11^,^31^. Effector Tfh cells have been demonstrated to express a high level of BCL-6; but in the memory phase, Tfh cells strongly down-regulate BCL-6 expression, although its expression is still higher than in memory non-Tfh or naive CD4^+^ T cells^32^,^33^. Consistent with these previous studies, we observed that the CD4^+^CXCR5^hi^PD-1^hi^ Tfh cells residing at the UP, which have a memory phenotype, still express a higher level of BCL-6 than CD4^+^CXCR5^lo^PD-1^lo^ non-Tfh cells (Fig. 3); and the UP-derived CD4^+^ T cells, which contain a large part of memory Tfh cells, show a non-significant increase of BCL-6 expression compared with those derived from the PB and SP (Figs 1, 2, 3).

The fundamental function of Tfh cells is to provide cognate help to B cells^11^. Recently, Tfh cells have been demonstrated to be involved in orchestrating alloimmune response and mediating allograft rejection, which are the major obstacles to clinical transplantation^13^,^14^,^34^. In a heart transplantation model, Tfh cells were found to help germinal centre alloantibody responses by an indirect-allorecognition pathway^35^. After allogeneic hematopoietic stem cell transplantation, Tfh cells were proven to be increased and required for the development of chronic graft-versus-host disease^15^. In addition, in the patients with chronic renal allograft rejection, increased circulating Tfh-cell ratio and decreased PD-1 expression were observed^36^. Furthermore, in the kidney biopsies taken during rejection, researchers found that the Tfh cells co-localized with CD20^+^ B cells and immunoglobulins in follicular-like structures, suggesting that Tfh cells might act as a bridge between cellular and humoral reactivity in acute rejection of kidney transplantation^37^.

The development of a semi-allogeneic foetus in the mother is also considered to be a physiological model of *in-vivo* allograft transplantation^38^,^39^. Data in our present study indicated that Tfh cells might be involved in the maintenance of an allogeneic pregnancy. We observed that UP-derived CD4^+^ T cells display Tfh-like and memory/activation phenotypes (Fig. 1), and a remarkably higher proportion of Tfh cells is accumulated in the UP compared with other lymphoid tissues (Fig. 2). On the other hand, increased foetal resorption by PDL1 blockade correlated with enhanced accumulation of Tfh cells (Figs 6 and 7 and Supplementary Fig. 6). Thus, our findings indicated that appropriate accumulation of Tfh cells during gestation might help to maintain a successful pregnancy, whereas an excessively high level of these cells could lead to abortion.

PD-1, with its ligands, PDL1 and PDL2, delivers inhibitory signals to regulate the equilibrium among T-cell activation, tolerance, homeostasis and immunopathology^40^. Recently, PD-1–PDL1 negative costimulatory pathway was proven to be critical to induce foeto-maternal tolerance and maintain normal pregnancy, by reducing CD4^+^ Tregs, but favoring Th1- and Th17-cell development^21^,^22^,^23^. In this study, we demonstrated that PDL1 blockade leads to increased foetal resorption through another possible mechanism: by favoring Tfh-cell development and upregulating ICOS and PD-1 expression (Figs 6, 7, 8 and Supplementary Figs S6 and 7). Our data, together with other findings^21^,^22^,^23^, suggested that during an allogeneic pregnancy, the Th1/Th2/Th17/Treg paradigm should be developed into a new Th1/Th2/Th17/Treg/Tfh paradigm.

Patrice Nancy and colleagues revealed that at mid-pregnancy of allogeneic mated mice (E10.5), a large number of CD3^+^ T cells distributed throughout the myometrium and endometrium of uterine segments, but appeared very sparse in the placenta and decidua, which was partly attributed to the epigenetic silencing of the key T cell–attracting inflammatory chemokine genes in DSCs^38^. However, Mohamed Habbeddine *et al.* showed that comparable numbers of CD4^+^ and CD8^+^ T cells resided in the placenta and uterus, at late-pregnancy of allogeneic mated mice (E16.5)^41^. Our results showed that the CD4^+^CXCR5^hi^PD-1^hi^ and CD4^+^CXCR5^hi^ICOS^hi^ Tfh cells, known as a subset of CD4^+^ T cells, were almost totally located in the uterus at mid-pregnancy (E11.5), but greatly increased in the placenta at late-pregnancy (E18.5), in the allogeneic pregnant mice (Fig. 5). Furthermore, Indira Guleria *et al.* observed that the CD3^+^ T cells were greatly infiltrated in the placenta as early as on E10.5 in the anti-PDL1 mAb-treated allogeneic pregnant mice^21^. In the present study, increased foetal resorption by PDL1 blockade correlated with enhanced accumulation of Tfh cells residing at the UP (Figs 6 and 7); however, whether PDL1 blockade could affect the location of Tfh cells and facilitate Tfh-cell infiltration into the placenta (and/or uterus) to increase the foetal resorption requires further investigation.

In summary, our study is the first to characterize the Tfh cells in allogeneic normal pregnancy and PDL1 blockage-induced abortion. We demonstrated that the CD4^+^ T cells residing at the UP display a Tfh-like phenotype, and the UP-derived CD4^+^CXCR5^hi^PD-1^hi^ and CD4^+^CXCR5^hi^ICOS^hi^ Tfh cells, which show high levels of Tfh-associated molecules and display a memory/activation phenotype, are dominantly enriched at mid-pregnancy. In addition, these Tfh cells are located abundantly in the uterus at mid-pregnancy, but greatly increased in the placenta at late-pregnancy. Moreover, increased foetal resorption by PDL1 blockade correlates with enhanced Tfh-cell accumulation and upregulated ICOS and PD-1 expression on these cells. Together, these findings deepen our understanding of the immunological events during pregnancy and provide novel insights into the physiology of normal pregnancy and the pathogenesis of abortion. Further studies are needed to investigate the precise locations (where Tfh cells are positioned in the uterus and placenta), definitive roles and underlying mechanisms of Tfh cells in normal and complicated pregnancies, and to explore novel approaches to target the immune cells to treat human miscarriage.

## Materials and Methods

### Mice

Female BALB/c (H-2^d^) and male C57BL/6 (H-2^b^) mice at 8–10 weeks of age were obtained from the Shanghai Laboratory Animal Center, Chinese Academy of Science, and subsequently housed under specific pathogen-free conditions in the Department of Laboratory Animal Science, Shanghai Jiaotong University School of Medicine (Shanghai, China). To establish an allogeneic-normal-pregnant animal model, female BALB/c mice were mated with male C57BL/6 mice and embryonic day 0.5 (E0.5) was defined as the day of vaginal-plug discovery. All animal experiments were approved and performed in compliance with the animal care and use committee guidelines of Shanghai Jiaotong University School of Medicine (Shanghai, China).

### Preparation of mononuclear cells

Mice were anaesthetized by intraperitoneal injection of pentobarbital sodium (50 mg/kg body-weight). To obtain peripheral blood mononuclear cells (PBMCs), blood samples were harvested from the vena orbitalis, and collected in phosphate buffered saline (PBS) with 2% EDTA. PBMCs were isolated using density gradient centrifugation with Lymphoprep^TM^ (AS1114546, Axis-shield). Isolations of mononuclear cells in the uterus and/or placenta were performed as previously described^41^,^42^. Briefly, after performing hysterolaparotomy and removing the embryos carefully, the uterus (containing mesometrial triangle) and/or placenta tissues were cut into small pieces (<1 mm^3^) using ocular scissors and filtered to obtain a single cell suspension. Mononuclear cells were also isolated and purified by density gradient centrifugation using Lymphoprep^TM^. To harvest splenic and thymic mononuclear cells, the whole spleen and thymus were disrupted mechanically and gently using a syringe (5 mL) plunger, and passed through a 70-μm cell strainer (352350, BD Biosciences). Red blood cells were eliminated through incubation with ACK Lysing Buffer (A10492-01, Gibco) for 1 minute, and the splenic and thymic mononuclear cells were harvested by centrifugation. Total number of mononuclear cells in the spleen and thymus was determined using a Leica DMI 3000B microscope with trypan blue exclusion. Bone marrow-derived mononuclear cells were isolated from femurs, and harvested by centrifugation.

### Treatment protocol

Pregnant females were injected intraperitoneally with the anti-mouse PDL1 blocking mAb (clone: 10F.9G2, BioLegend) at a dose of 250 μg on E5.5 and E8.5, respectively. Pregnant mice were sacrificed on E11.5, and the embryo-resorption rate was determined as previously described^43^, and calculated as follows: Resorption rate = (number of resorbed embryos/number of total embryos) × 100.

### mAbs and reagents

Fluorescein-conjugated mAbs, including anti-CD4-Pacific Blue (clone: RM4-5), anti-CD8- APC-Cy7 (53-6.7), anti-CXCR5-PE-Cy7 (2G8), anti-CD44 (Pgp-1/Ly-24)-V500 (IM7) and anti-CD152 (CTLA-4)-PE (UC10-4F10-11) were purchased from BD Pharmingen; anti-CD278 (ICOS)-PE-Cyanine5 (7E.17G9), anti-CD154 (CD40 Ligand)-PE-Cyanine7 (MR1) and anti-CD127-PerCP-Cyanine5.5 (A7R34), were from eBioscience; and anti-CD279 (PD-1)-PE (RMP1-30) and anti-BCL-6-APC (7D1) were from BioLegend.

### Flow cytometry

Surface staining and intranuclear transcription factor staining were performed as previously described^44^,^45^. Briefly, isolated mononuclear cells were incubated with different cocktails of mAbs for surface antigens in 100 μL PBS containing 3% (v/v) FBS at room temperature for 30 minutes. Intranuclear transcription factor staining was performed according to the Foxp3 staining protocol (00-5523-00, eBioscience) after staining with the indicated surface mAbs. Cells were treated with Fixation/Permeabilization Buffer for 1 hour, and subsequently incubated with anti-BCL-6 mAb for 40 minutes at room temperature. Immunostained cells were collected and analysed on a BD FACS Canto II flow cytometer (BD Biosciences, USA). Data were processed using the FlowJo 7.6.1 software.

### Statistical analyses

All experiment results are shown as means ± standard error of means^42^. The Kolmogorov-Smirnov test was used to evaluate the normality of the data. If the data were normally distributed, statistical analysis was performed using unpaired or paired Student’s *t*-test, or one-way ANOVA followed by Tukey’s post-tests; otherwise, the Mann-Whitney U or Wilcoxon matched pairs test, or Kruskal-Wallis test followed by Dunns multiple-comparison test, was performed. These are described in detail in the Figure Legends. *P-*values < 0.05 were considered statistically significant.

## Additional Information

**How to cite this article**: Zeng, W. *et al.* Characterization of T follicular helper cells in allogeneic normal pregnancy and PDL1 blockage-induced abortion. *Sci. Rep.* **6**, 36560; doi: 10.1038/srep36560 (2016).

**Publisher’s note:** Springer Nature remains neutral with regard to jurisdictional claims in published maps and institutional affiliations.

## Supplementary Material

Supplementary Information
