# Supplementary material for: Characterization of T follicular helper cells in allogeneic normal pregnancy and PDL1 blockage-induced abortion
Source: Sci Rep. 2016 Nov 7;6:36560. doi: 10.1038/srep36560 (PMC5098204; doi:10.1038/srep36560)
Supplement: Supplementary Information [file srep36560-s1.pdf]

Title page

Title: Characterization of T follicular helper cells in allogeneic normal pregnancy and PDL1 blockage-induced abortion

Authors:

Weihong Zeng[1], Zhicui Liu[2], Siming Zhang[1], Jiabin Ren[3], Xiaoling Ma[1], Chuanmei Qin[1], Fuju Tian[1], Yan Zhang[4, *] and Yi Lin[1, *]

Institution:

[1] Institute of Embryo-Fetal Original Adult Disease Affiliated to Shanghai Jiao Tong University School of Medicine, the International Peace Maternity & Child Health Hospital, Shanghai Jiao Tong University School of Medicine, Shanghai 200030, P. R. China.

[2] Department of Dermatology, Ruijin Hospital, Shanghai Jiaotong University School of Medicine, Shanghai 200025, P. R. China.

[3] Department of Obstetrics and Gynecology, Ren Ji Hospital, Shanghai Jiaotong University School of Medicine, Shanghai 200127, P. R. China.

[4] Department of Obstetrics and Gynecology, Renmin Hospital of Wuhan University, Wuhan 430060, P. R. China.

* **Corresponding author:**

Yan Zhang, Department of Obstetrics and Gynecology, Renmin Hospital of Wuhan University, Wuhan 430060, P. R. China. Telephone: +86-27-88041911. Fax: +86-27-88041911. E-mail: zyan2200@gmail.com.

Yi Lin, Institute of Embryo-Fetal Original Adult Disease Affiliated to Shanghai Jiao Tong University School of Medicine, the International Peace Maternity & Child Health Hospital, Shanghai Jiao Tong University School of Medicine, No. 910, Hengshan Road, Shanghai 200030, P. R. China. Telephone: +86-21-64070434. Fax: +86-21-64073421. E-mail: yilinonline@126.com.

Supplemental Materials

Material and Method

ELISA

CXCL13 level in murine serum was determined by using a Mouse CXCL13 ELISA Kit (EK0740, BOSTER) according to the manufacturer’s instruction.

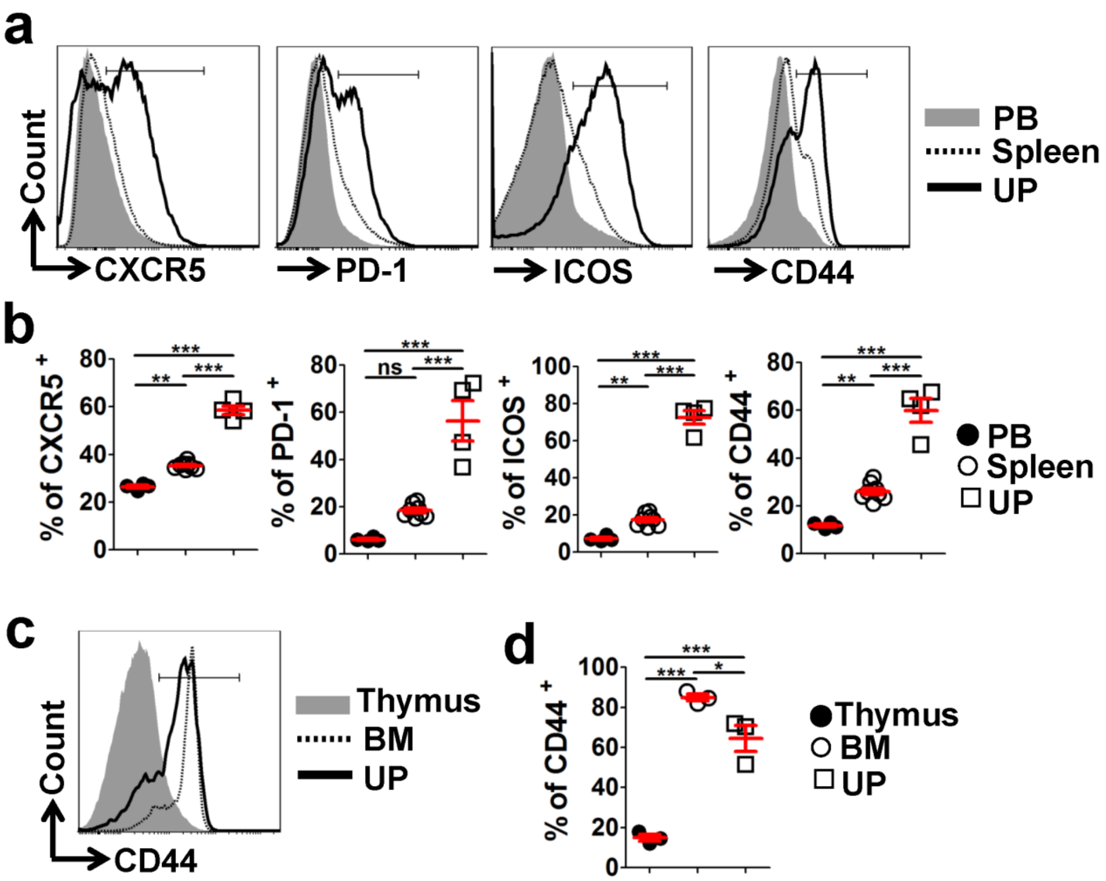

Figure S1. CD4$^+$ T cells residing at the UP display Tfh-like and memory/activation phenotypes at mid-pregnancy. (a-d) Representative flow cytometric histograms (a and c) and cumulative data (b and d) illustrating the percentages of the indicated proteins among CD4$^+$ T cells in the PB, spleen and UP (a-b), as well as in the thymus, BM and UP (c-d), of maternal mice on E11.5. Each symbol reflects the data from a single mouse ($n \geq 3$ mice per group) and the data are representative of at least three independent experiments. The cells are gated in CD4$^+$ T cells. Data were assessed statistically using one-way ANOVA followed by Tukey's post-tests. PB: peripheral blood; UP: uterus and placenta; BM: bone marrow; ns: not significant; *: $p < 0.05$; **: $p < 0.01$; ***: $p < 0.001$.

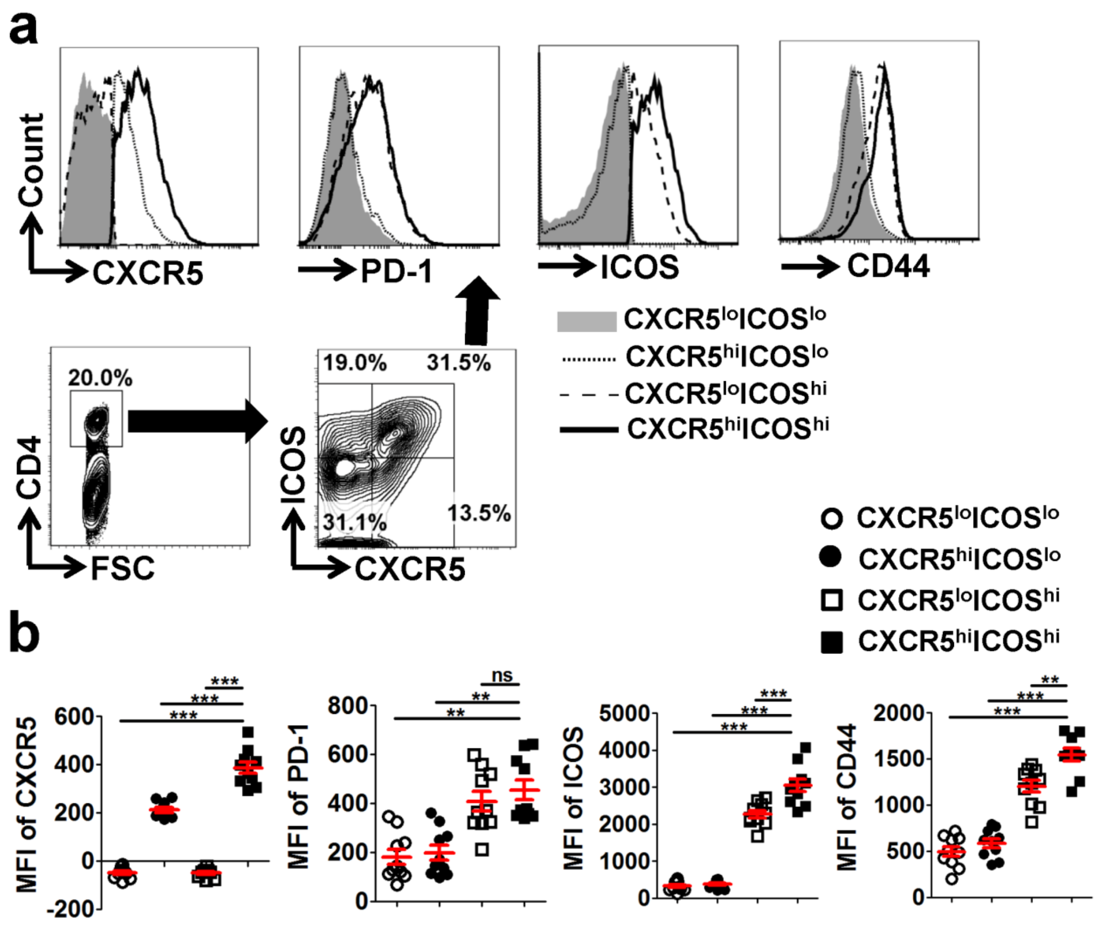

Figure S2. CD4^{+}CXCR5^{hi}ICOS^{hi} Tfh cells residing at the UP show high levels of Tfh-associated molecules and display a memory/activation phenotype. (a) CD4^{+} T cells were gated from the lymphocytes in the UP of maternal mice on E11.5, and a schematic diagram illustrates the strategy used to analyse the molecular expression of indicated proteins on CD4^{+}CXCR5^{lo}ICOS^{lo}, CD4^{+}CXCR5^{hi}ICOS^{lo}, CD4^{+}CXCR5^{lo}ICOS^{hi} and CD4^{+}CXCR5^{hi}ICOS^{hi} cells. **(b)** Comparisons of the molecular expression of CXCR5, PD-1, ICOS and CD44 on different CD4^{+} T-cell subsets derived from the UP of pregnant mice on E11.5. Each symbol reflects the data from a single mouse (n≥ 6 mice per group) and the data are representative of at least three independent experiments. Geometric MFI values were calculated using FlowJo

7.6.1 software and the data were assessed statistically using one-way ANOVA followed by Tukey's post-tests. UP; uterus and placenta; MFI: mean fluorescent intensity; hi: high; lo: low; ns: not significant; **: $p < 0.01$; ***: $p < 0.001$.

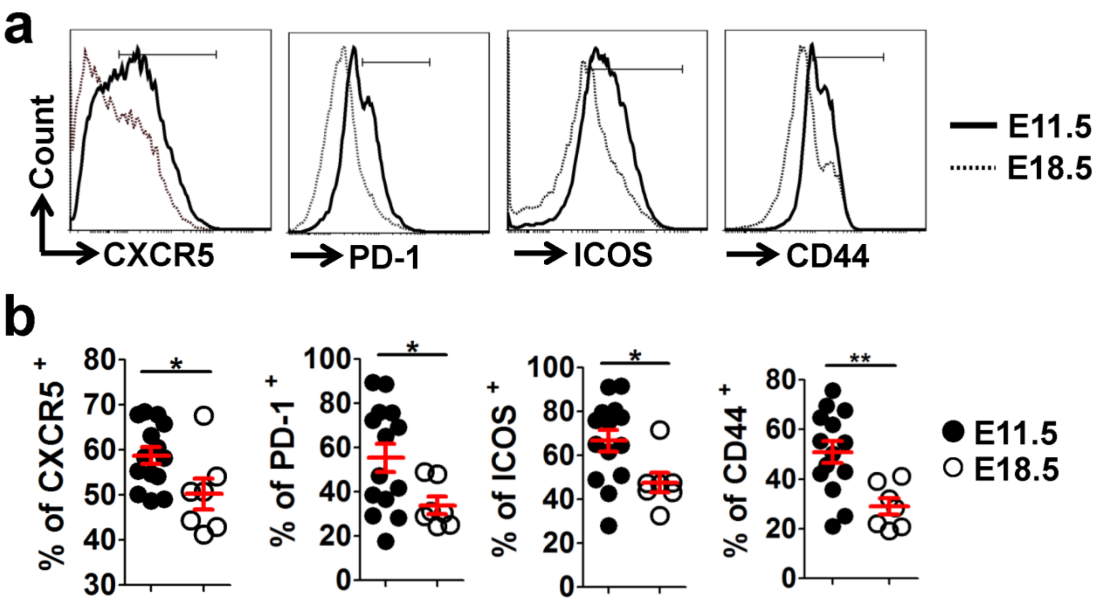

Figure S3. CD4+ T cells residing at the UP express higher levels of Tfh-associated molecules and memory/activation marker at mid-pregnancy. (a-b) Representative flow cytometric histograms (a) and cumulative data (b) illustrating the percentages of CXCR5, PD-1, ICOS and CD44 among CD4+ T cells derived from the UP on E11.5 and E18.5. Each symbol reflects the data from a single mouse ($n \geq 7$ mice per group) and the data are representative of at least two independent experiments. The cells are gated in CD4+ T cells and the statistical analysis was performed using the Mann-Whitney U test. UP: uterus and placenta; *: $p < 0.05$; **: $p < 0.01$.

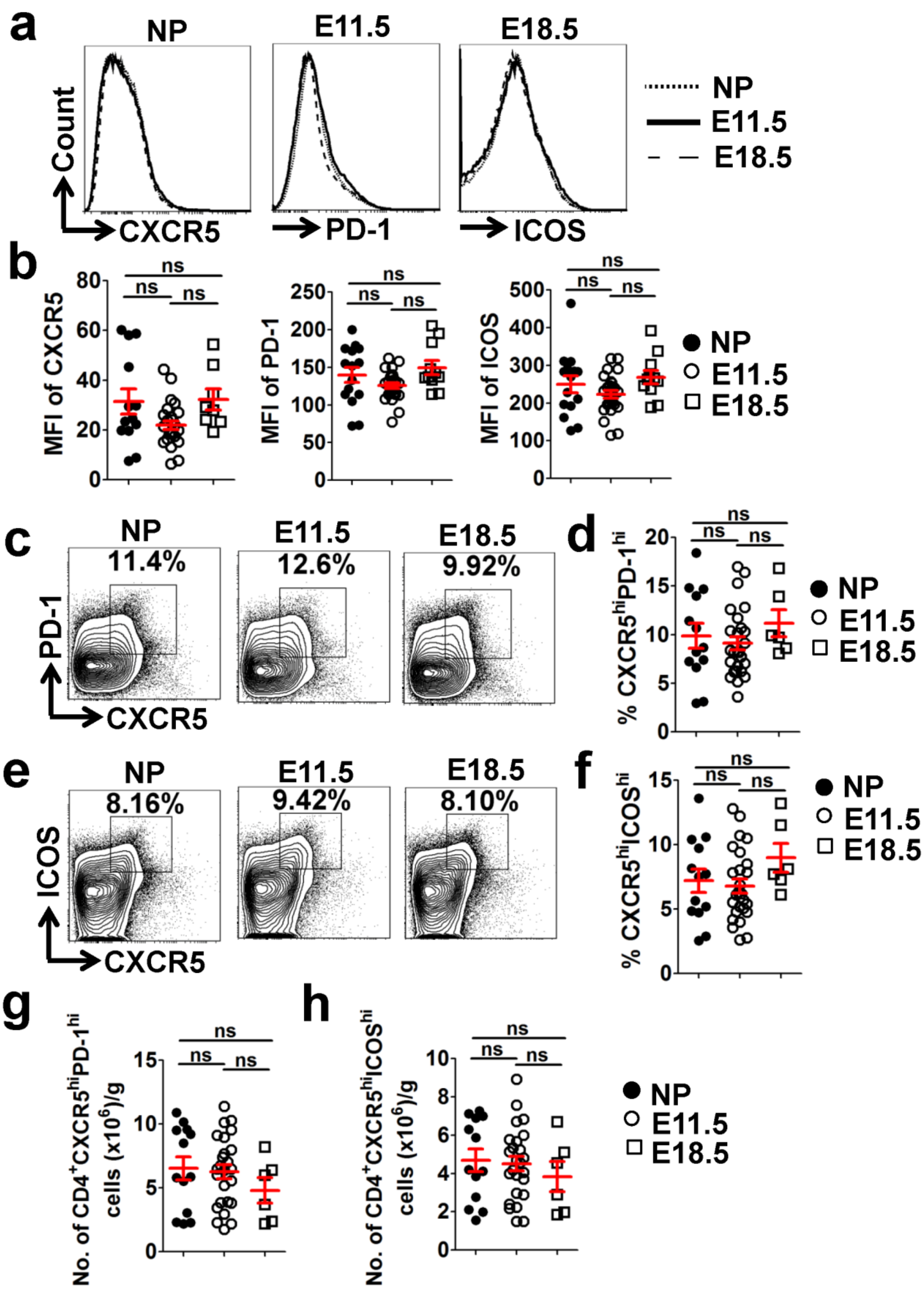

Figure S4. Dynamic changes of Tfh-associated molecular expression on CD4$^+$ T cells, as well as the proportion and concentration of Tfh cells, in the maternal spleen during pregnancy. (a-f) Representative flow cytometric histograms (a) or plots (c and e) and cumulative data (b, d and f) illustrating the molecular expression of

CXCR5, PD-1 and ICOS (a-b), as well as the percentages of $CXCR5^{hi}PD\text{-}1^{hi}$ and $CXCR5^{hi}ICOS^{hi}$ populations (c-f), among the $CD4^+$ T cells in the maternal spleen during pregnancy. **(g-h)** Absolute Tfh-cell numbers per gram in the maternal spleen during pregnancy. Each symbol reflects the data from a single mouse ($n \geq 6$ mice per group) and the data are representative of at least two independent experiments. The cells are gated in $CD4^+$ T cells. Geometric MFI values were calculated using FlowJo 7.6.1 software and the data were assessed statistically using one-way ANOVA followed by Tukey's post-tests. NP: non-pregnant; MFI: mean fluorescent intensity; hi: high; No.: number; ns: not significant; *: $p < 0.05$; **: $p < 0.01$.

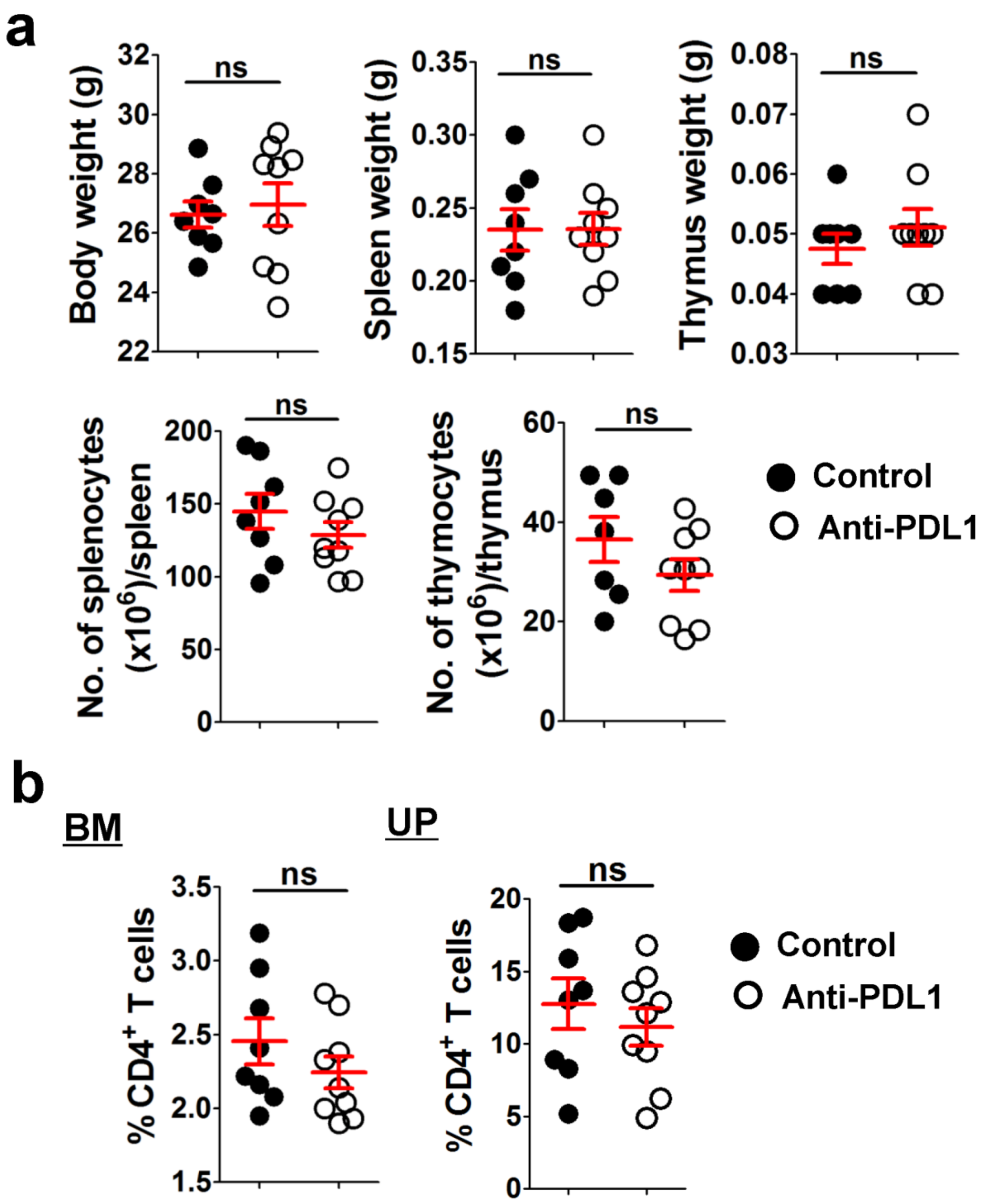

Figure S5. The body-, spleen-, and thymus-weight, and the absolute number of splenocytes and thymocytes, together with the proportion of $CD4^+$ T cells, are not affected by PDL1 blockade. (a) Comparisons of the body-, spleen-, and thymus-weight, as well as the absolute number of splenocytes and thymocytes, between the control and PDL1-blocked mice. **(b)** Comparisons of the proportion of $CD4^+$ T cells in the BM and UP between the control and PDL1-blocked mice. Each

symbol reflects the data from a single mouse ($n \geq 7$ mice per group) and the data are combined from four independent experiments. The data were assessed statistically using an unpaired Student's *t*-test. No.: number; BM: bone marrow; UP: uterus and placenta; ns: not significant.

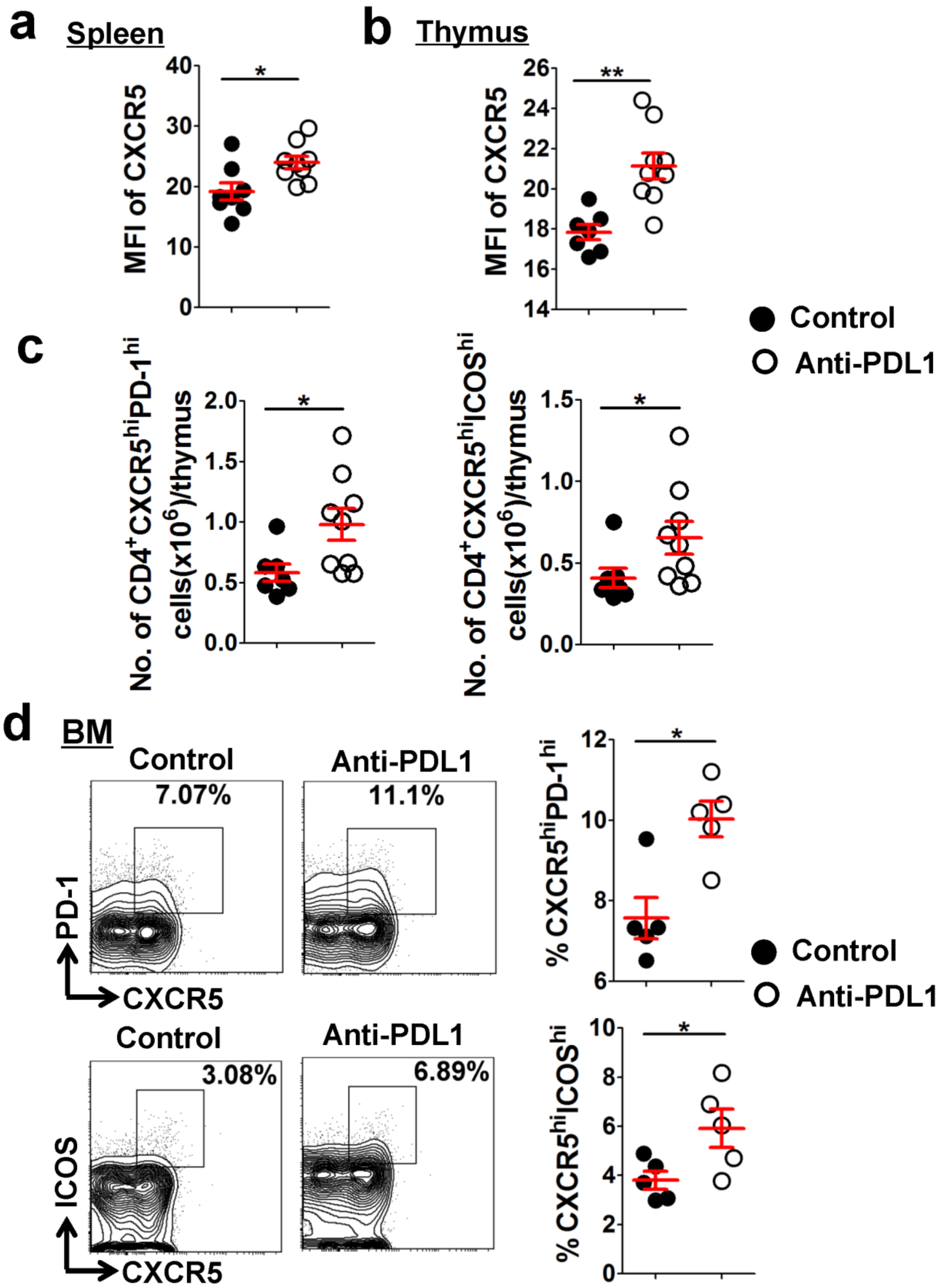

Figure S6. PDL1 blockade upregulates CXCR5 expression on CD4$^+$ T cells, and increases the proportion and cellularity of Tfh cells. (a-c) Comparisons of CXCR5 expression on the splenic or thymic CD4$^+$ T cells (a-b), and the absolute number of CD4$^+$CXCR5^{hi}PD-1^{hi} or CD4$^+$CXCR5^{hi}ICOS^{hi} Tfh cells in the maternal thymus (c),

between the control and PDL1-blocked mice. **(d)** Representative flow cytometric plots and cumulative data illustrating the percentages of CXCR5^{hi}PD-1^{hi} and CXCR5^{hi}ICOS^{hi} populations among CD4^{+} T cells in the maternal BM of the control and PDL1-blocked mice. Each symbol reflects the data from a single mouse ($n \geq 5$ mice per group). Geometric MFI values were calculated with FlowJo 7.6.1 software and the data were assessed statistically using an unpaired Student's *t*-test (a-b) or the Mann-Whitney U test (c-d). MFI: mean fluorescent intensity; No.: number; hi: high; BM: bone marrow; *: $p < 0.05$; **: $p < 0.01$.

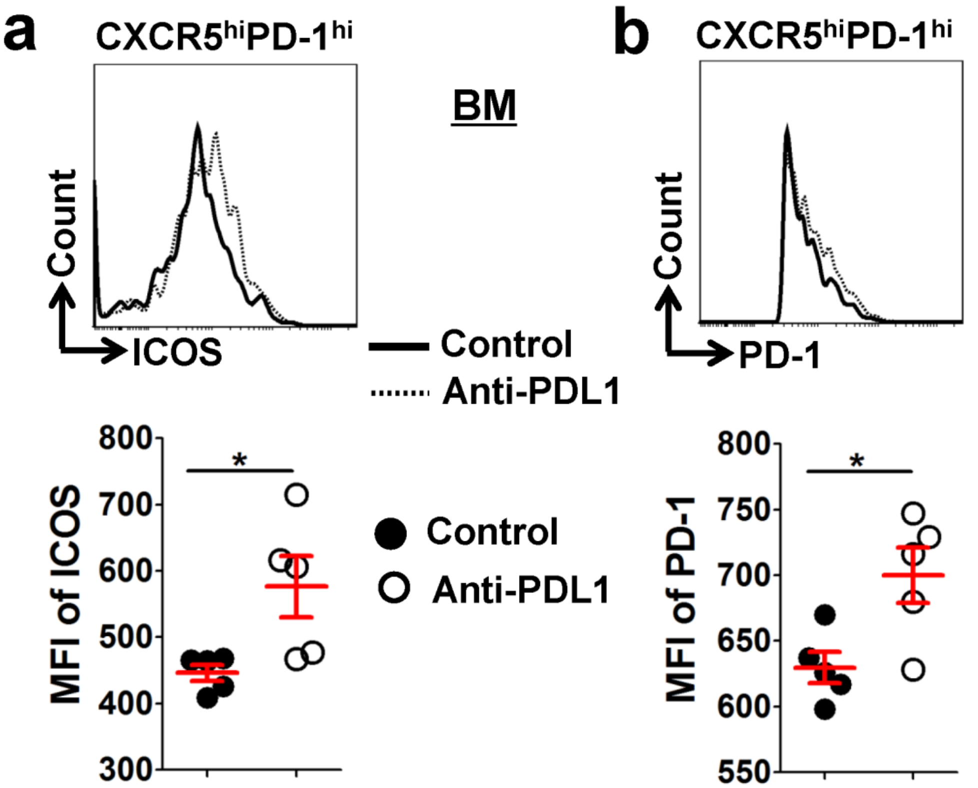

Figure S7. PDL1 blockade upregulates ICOS and PD1 expression on Tfh cells in the maternal BM. **(a-b)** Representative flow cytometric histograms and cumulative data illustrating ICOS (a) and PD-1 (b) expression on $CD4^{+}CXCR5^{hi}PD\text{-}1^{hi}$ Tfh cells from the maternal BM. Each symbol reflects the data from a single mouse (n≥ 5 mice per group). Geometric MFI values were calculated using FlowJo 7.6.1 software and the data were assessed statistically using an unpaired Student's *t*-test. BM: bone marrow; hi: high; MFI: mean fluorescent intensity; *: $p < 0.05$.

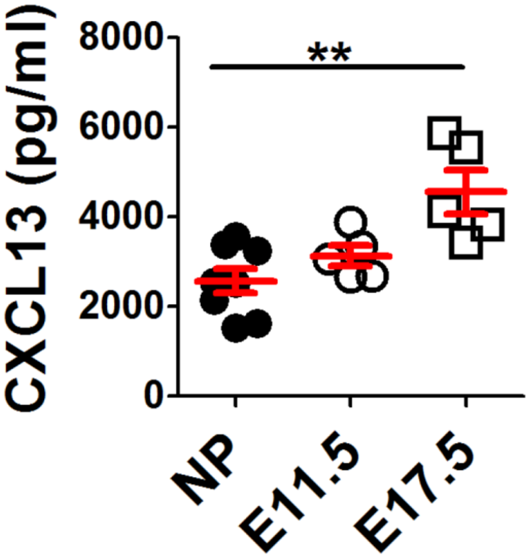

Figure S8. The CXCL13 level in murine serum was greatly elevated during allogeneic normal pregnancy. Sera were harvested from C57BL/6-mated BALB/c pregnant mice on E11.5 and E17.5, or from NP BALB/c females. The CXCL13 level in serum was determined using a Mouse CXCL13 ELISA Kit. Each symbol reflects the data from a single mouse (n ≥ 5 mice per group) and the data were assessed statistically using one-way ANOVA followed by Tukey's post-tests. NP: non-pregnant; **: $p < 0.01$.
